# Evaluating the financial protection of patients with chronic disease by health insurance in rural China

**DOI:** 10.1186/1475-9276-8-42

**Published:** 2009-12-09

**Authors:** Qiang Sun, Xiaoyun Liu, Qingyue Meng, Shenglan Tang, Baorong Yu, Rachel Tolhurst

**Affiliations:** 1 Centre for Health Management and Policy, Shandong University, 44 Wenhua Xi Road, Jinan, 250012, Shandong, PR China; 2 Liverpool School of Tropical Medicine, Pembroke Place, Liverpool, L3 5QA, UK

## Abstract

**Background:**

A growing number of developing countries are developing health insurance schemes that aim to protect households, particularly the poor, from financial catastrophe and impoverishment caused by unaffordable medical care. This paper investigates the extent to which patients suffering from chronic disease in rural China face catastrophic expenditure on healthcare, and how far the New Co-operative Medical Insurance Scheme (NCMS) offers them financial protection against this.

**Methods:**

A household survey was conducted in six counties in Ningxia Autonomous Region and Shandong Province, with a total of 6,147 rural households, including 3944 individual chronic disease patients. Structured questionnaires were used with chronic disease patients to investigate: their basic social and economic characteristics, including income and expenditure levels and NCMS membership; and their health care utilization, associated healthcare costs and levels of reimbursement by NCMS. 'Catastrophic' expenditure was defined as healthcare expenditure of more than 40% of household non-food expenditure.

**Results:**

Expenditure for chronic diseases accounted for an average of 27% of annual non-food per capita expenditure amongst NCMS members in Shandong and 35% in Ningxia. 14-15% of families in both provinces spent more than 40% of their non-food expenditure on chronic healthcare costs. Between 8 and 11% of non NCMS members and 13% of NCMS members did not seek any medical care for chronic illness. A greater proportion of NCMS members in the poorest quintile faced catastrophic expenditure as compared to those in the richest quintile in both study sites. A slightly higher proportion of non-NCMS members than NCMS member households faced catastrophic expenditure, but the difference was not statistically significant.

**Conclusion:**

A significant proportion of patients with chronic diseases face catastrophic healthcare costs and these are especially heavy for the poor. The NCMS offers only a limited degree of financial protection. The heavy financial burden of healthcare for chronic disease poses an urgent challenge to the NCMS. There is an urgent need for a clear policy on how to offer financial protection to those with chronic disease.

## Background

### China's rural health insurance in the international context

A growing number of developing countries are developing health insurance schemes that aim to protect households, particularly the poor, from financial catastrophe and impoverishment caused by unaffordable medical care. China, in common with other transitional economies, has experienced rapid economic and healthcare reforms over the past two decades. As a result of these reforms, the majority of households living in rural China face out of pocket charges for healthcare that are rising faster than their incomes. Prior to the reforms, over 90% of the rural Chinese population were covered by Co-operative Medical Schemes (CMS), which collapsed in the mid 1980s. In recent years the Chinese government has been reinstating health insurance, now known as the New Rural Co-operative Medical Scheme (NCMS).

There is a wide diversity of health insurance schemes in developing countries, ranging from 'community-based' schemes, which are often voluntary, mainly funded by the households and local communities, with limited government support, to national schemes with substantial government support, which aim towards comprehensive coverage, as is the case in China, although participation in the scheme is voluntary [1-3]. In theory, the primary purpose of any insurance scheme is to share risk between individuals and hence extend financial protection to members of the scheme [4]. The central stated goal of developing the rural health insurance scheme in China is to provide financial protection to individuals and households from catastrophic expenditures due to major illness [5].

In principle financial protection is achieved through pooling the contributions of all members and pooling risks to redistribute them horizontally between healthy and sick and vertically between richer and poorer members of the scheme. However, lack of cross-subsidisation across communities and inadequate cross-subsidisation within communities due to low coverage levels and adverse selection have been common reasons for scheme failure [6,7]. Low coverage has resulted from a range of factors, including low capacity to pay premium contributions [1]. In all countries with (nearly) universal coverage, health care or insurance contributions of the poor are subsidized or directly paid by the government [3].

The NCMS has made rapid progress in increasing coverage to high levels since its inception in 2004. Recent estimates of the national average enrolment level have ranged from 82.7% [8] and 85.7% [9]. A high level of political commitment has been an important factor enabling this remarkable achievement [9]. Subsidies from national and local governments have enabled a relatively low individual premium level, which contributes to the high coverage rate. Premiums for the poorest are paid directly through by the government through the Medical Financial Assistance Scheme for the Poor [10].

The coverage of catastrophic risk versus primary healthcare services is a major dilemma facing many schemes. Given limited funding and the goal of protecting households from medical impoverishment, the majority of schemes focus on providing insurance against "catastrophic" inpatient expenses, with the rationale that most households can afford the expenses incurred by minor illnesses [11]. However, there is increasing empirical evidence that household spending on ambulatory services and drugs, rather than hospitalization, is the primary contributor to medical impoverishment [11-15]. The under-utilization of primary care, and higher costs related to the irrational utilization of hospital facilities for illnesses treatable at lower levels of the health system has also been recognised as a factor influencing the failure of some schemes [12]. Yip and Hisao [12] argue that coverage of primary care and outpatient services provides incentives for patients to use these levels of care, rather than hospital services.

Less than optimal mechanisms for provider payment (such as fee-for-service systems) resulting in supplier-induced demand have posed significant challenges to many HI schemes [1,16-18]. Rapid increases in health expenditure in China in recent years have been linked to the fee-for-service payment modality and the need to control the high cost of healthcare has been an increasing concern in NCMS implementation [18-20].

### Non-communicable disease burden in China

Chronic diseases account for an estimated 80% of total deaths and 70% of total disability-adjusted life-years (DALYs) lost in China [21]. According to the third National Health Services Survey in 2003, the morbidity rate of chronic diseases in the rural areas was 10.5%, and the most common diseases were: hypertension, gastroenteritis, rheumatoid Arthritis, chronic obstructive pulmonary disease (COPD), cerebrovascular disease, inter-vertebral disease disorders and peptic ulcers [22] (Ministry of Health 2004). Chronic diseases have become the leading cause of death in both urban and rural areas, with mortality rates of 85.3% and 79.5% respectively [23].

In 2003, the estimated economic burden of chronic diseases in China accounted for 71.5% of the total economic burden of all diseases and 7.3% of GDP [24]. The five diseases with the highest economic burden were malignancy, cerebrovascular disease, hypertension, coronary heart disease, and other types of heart diseases, accounting for 39.6% of the total economic burden of all chronic non-communicable diseases and 28.2% of the total economic burden of all diseases (including the direct health care expense and indirect expense that is the foregone income due to illness) [24].

An estimated 54% of total medical expenditure (by households) on chronic diseases was spent on outpatient services, rising to 67% for hypertension and 74% for rheumatoid arthritis [24]. Average inpatient expenditure on common chronic diseases, such as hypertension and diabetes, accounts for 1.5 times the annual per capita income of rural residents [25]. One study in Shandong and Gansu provinces estimated the annual per capita outpatient expenditure on chronic diseases at half of total annual medical expenditure [26]. The available evidence therefore suggests that chronic diseases pose a heavy financial burden for rural residents, and that outpatient expenditure is a significant part of this burden.

### NCMS development

The New Cooperative Medical Scheme (NCMS) was introduced on a pilot basis in 2003 and had expanded to 86% of China's rural counties by 2007, covering about 730 million rural people [26-29]. A key characteristic of NCMS is the increasing level of subsidies to the scheme from the Chinese government, with the aim of making the scheme fairer and financially more attractive to low-risk households. However, the 50 Yuan minimum level of financing per beneficiary represents only around one fifth of average per capita total health spending in rural areas [9] (at the time of data collection in May 2006, 1 US$ was equal to 8 Yuan). The new scheme operates at the county level, where there is considerable discretion in the design, including the benefit package, payment methods and reimbursement level.

As a result of the limited financial envelope, many services, and particularly outpatient care, are not covered or covered only partially, deductibles are high, ceilings are low, and coinsurance rates are high. There is, however, considerable heterogeneity in the benefit package across counties and coverage modes. One study of the 189 NCMS pilot counties in 2005, found that all counties covered inpatient care, only a quarter of counties covered outpatient expenses on a pooling basis, the rest did not cover them at all (10% of counties), covered only catastrophic expenses (10% of counties), or covered them through a so-called 'family account', which is not a pooled fund [9]. The bulk of reimbursement by NCMS is for inpatient expenses, even in counties where outpatient expenses are covered, and there are no special arrangements for reimbursing medical expenses resulting from the chronic diseases.

Since the establishment of NCMS in 2003, no study to our knowledge has investigated how far the new scheme contributes to alleviating the financial burden of chronic diseases. This paper aims to contribute to filling this gap by evaluating how far NCMS offers financial protection to rural residents with chronic diseases, particularly the poor, in Shandong Province and Ningxia Hui Nationality Autonomous Region, through an analysis of household survey data on healthcare utilization, costs and reimbursement by NCMS. Specifically the paper aims to answer the following research questions: 1) What is the financial burden of chronic disease in the study areas, especially for the poor? 2) How far does NCMS offer financial protection against chronic disease burdens, especially for the poor?

### NCMS in Shandong and Ningxia

This study was conducted in Shandong province and Ningxia Hui Nationality Autonomous Region. Shandong Province, which is located in the Eastern coastal area, represents the more economically developed regions of China, with a GDP per capita of 23,546 Yuan in 2006 (about 3139.5 US$). Ningxia Hui Nationality Autonomous Region, a relatively poor area in north western China, represents the less developed inland regions. The region has a GDP per capita of 11,784 Yuan (about 1571.2 US$), and 36% of its population are minority ethnic groups.

Shandong Province was included in the NCMS pilot of 2003, and all 134 counties are now covered by the NCMS, with about 59 million rural residents enrolled in the new scheme, an enrollment rate of 90.08%. The financing level per beneficiary varied in different counties, with 81% of 134 counties reaching 50-55 Yuan per beneficiary in 2007, and other counties exceeding this level, with the highest at 100 Yuan per beneficiary (maintaining the 10 Yuan contribution per rural resident). The benefit package varied across counties, but generally inpatient services were covered by risk pooling in all counties. Some counties reimbursed outpatient expenditure through risk pooling, and others set up so-called 'family accounts', whereby a fixed amount of the per capita financing is allocated to each household to cover outpatient expenditure; in 2005 this was 8 Yuan out of the 10 Yuan premium per person. This is a pre-payment rather than a risk pooling mechanism, but households can pool their allocations and accounts 'roll over' from year to year so funds can be accumulated. Once the fixed sum has been exhausted there is no further reimbursement for outpatient services available. However, a few counties reimbursed outpatient medical expenditure for some chronic diseases through a risk pooling mechanism. Meanwhile a combination of deductibles, co-payment, ceilings, an essential medicine list and an essential medical services list were used to control the health expenditure. Reimbursement is very complicated, with variation in rates according to the level of health facilities, the type of services (outpatient or inpatient) and the level of medical expenditure. Information provided by the Shandong Provincial Department for Health showed that in general, the reimbursement rate for outpatient services ranged from 10-20%, and inpatient services from 10-80%. In October 2007, about 48% of enrollers were reimbursed by NCMS, and the actual reimbursement rate of outpatient and inpatient expenditure was 27.99% and 25.07% respectively. The average reimbursement of inpatient medical expenditure was 616 Yuan.

Ningxia was also included in the NCMS pilot of 2003. By 2007, all 18 counties were covered by the scheme, and the enrollment rate was 85.05%. At present the financing level per beneficiary was 50 Yuan, including 20 Yuan from the central government, 20 Yuan from the local government and 10 from individual members. The benefit package of NCMS has been uniform in Ningxia since 2006, when all the counties set up 'family accounts' for reimbursing outpatient expenditure and a risk pooling mechanism for reimbursing inpatient expenditure. According to the Ningxia Autonomous Regional Department for Health, for inpatient services, the reimbursement level varies according to the level of health institution and medical expenditure, ranging from 40-50% at the township health center, to 35-45% at county hospital and higher levels, based on the amount of medical expenditure. The real reimbursement rate at county hospital is currently about 35%, and 45% at township health center. Deductibles, co-payment, ceilings, an essential medicine list and an essential medical services list were also used to control the health expenditure.

## Methods

### Study design and sampling

The data used in this paper are derived from a cross-sectional household survey conducted in Shandong and Ningxia in 2006, as part of a project aiming to improve the design of rural health insurance in rural China and Vietnam. The study is entitled 'Bringing health care to the vulnerable - developing equitable and sustainable rural health insurance in China and Vietnam (RHINCAV)' and is funded by the European Community. For further details please see http://www.liv.ac.uk/lstm/research/rhincav/index.htm. The selection of the two provinces aimed to represent different levels of socio-economic development and geographic areas in China. Three counties were selected from Shandong (Zhangqiu, Changle, and Dong'e) and three from Ningxia (Zhongning, Yongning and Qingtongxia). The selection of counties was based on the following criteria: 1) all the counties have an existing New Rural Cooperative Medical Scheme (NCMS); 2) the County governments are willing and able to collaborate with the research project; and 3) the three counties should have different levels of socio-economic development and be located in a similar geographic area.

The household survey used a multistage sampling process. Three townships in each county and three villages in each township were selected using similar criteria to the selection of counties. In each village, a random sample of 100 households was selected based on the household registration list. The calculation of the sample size was based on the ability to compare the utilization rate for impatient services among 5 different income groups. A total of 6,147 households and 22,636 individual rural residents were sampled and investigated. Among them, 3944 individual patients responded that they had suffered from a (self identified) chronic disease in the previous year (May 2005 - April 2006). This paper presents an analysis of the sub-database of chronic disease patients.

### Data collection and analysis

Structured questionnaires were used to conduct face to face interviews with people who reported suffering from one or more chronic diseases (in Chinese: *man xing bing*) to investigate: their basic social and economic characteristics, including income and expenditure levels and NCMS membership; and their health care utilization, associated healthcare costs and levels of reimbursement by NCMS where applicable. Master's students in Social Medicines were trained to conduct the questionnaire interviews. Data collected were for the past year - i.e. May 2005 to April 2006. Chronic disease was defined as an illness or symptom with at least 3 months continuous duration or intermittent presentation within the last 12 months. Interviewees were asked to give the name or main symptoms of each chronic disease they reported and, if relevant, where the disease was diagnosed (type and level of healthcare provider).

All the data were double entered in the database designed by Microsoft Office Access, and SPSS software was used to analyze the data. X^2 ^and T test were the main statistical methods used in the analysis for this paper.

To estimate the incidence of 'catastrophic' expenditure posed by chronic disease, we calculated the proportion of healthcare expenditure to household non-food expenditure. Estimates of expenditure were elicited from each household by asking the respondent to recall expenditure in response to a detailed list, encompassing production expenditure, consumption expenditure (including expenditure on: food, clothing and other daily living expenses; transportation and communication; housing, electricity, water and fuel; culture, education and entertainment; drugs, health care, and medical supplies) and other expenditures (e.g. paying back debt and interest, premiums, gifts, penalties and taxes). There is no international consensus on the threshold proportion of medical expenditure to household expenditure that constitutes 'catastrophic' expenditure. In previous studies, the threshold has varied from 5% to 40% of total household income [30-32]. We used a conservative threshold of 40% following Xu et al. [32].

### Ethical approval

Ethical approval was obtained from the Research Ethics Committee at the Liverpool School of Tropical Medicine in UK and at School of Public Health of Fudan University in China (Approval No. IRB# 06-04-0061).

### Limitations of the study methodology

The methodology used has several limitations for meeting our objectives, which are mainly due to the fact that the household survey was not designed solely to investigate the chronic disease burden. First, our estimations are based on self-reported chronic disease, rather than only those diagnosed by a physician. It is therefore possible that chronic illness and consequently its costs are either over or under-reported, due to the lack of a standard set of criteria used to distinguish chronic from acute illness. However, the question used to elicit self-reporting of chronic disease was drawn from the National Health Survey, which has been validated nationally. In addition, if we had included only diseases diagnosed by a physician, this could underestimate the disease burden given the barriers to accessing a diagnosis. Second, expenditure on healthcare for chronic disease was estimated by respondents over the year prior to the survey, which may have led to some recall bias or error. Third, it is difficult to assess the level of financial protection offered to NCMS members in comparison to non-members for several reasons. There are problems in interpreting our data on the difference between members and non-members in the proportion of medical expenditure to annual non-food expenditure in Shandong, because of skewed data due to very high expenditure in one case. There are further difficulties interpreting the data on the difference in levels of 'catastrophic expenditure' between members and non-members because of the confounding factors in terms of other differences between the two groups, such as income levels and chronic disease prevalence. Our paper has therefore focused on the degree of financial protection provided to NCMS members. Finally, it is not possible to definitively identify when costs are actually 'catastrophic' to households, since these are not always synonymous with high health-care costs [30]. However, even small costs for common illnesses can be financially disastrous for poor households [33]. Our analysis uses the term 'catastrophic' healthcare costs in line with norms in the international literature but we recognise that these costs are accurately termed 'potentially catastrophic'.

## Results

### Sample characteristics and disease prevalence

#### Basic demographic characteristics of rural residents with chronic diseases

Table 1 presents the basic demographic characteristics of the sample. In both Shandong and Ningxia, most of the sampled rural patients were enrolled in NCMS: 93% in Shandong and 87% in Ningxia. There was no significant difference in the gender distribution of NCMS members in either province, but in Shandong, a significantly higher proportion of male than female rural residents did not join NCMS. Nearly 60% of rural NCMS members with chronic diseases were 35-60 years old in the two study sites, whilst the proportion of NCMS members over 60 years old was 35% and 39% respectively in Shandong and Ningxia. In Shandong, the annual per capita income of NCMS members was about 5800 Yuan, which was slightly less than that of non-members, but in Ningxia, the situation is the reverse, with members having higher average income level. The difference in income between members and non-members was statistically significant difference in both provinces.

**Table 1:** The basic characteristic of rural residents with chronic diseases (year 2005)

	Shandong		Ningxia	
	
	NCMS^i^	Non-NCMS^ii^	NCMS	Non-NCMS
Total No. of patients reporting chronic disease	1767 (93%)	134(7%)	1773(87%)	270(13%)
Total No. of chronic cases^iii^	2191	164	2450	362
Gender				
Male	774 (44%)	92 (69%)*	736 (42%)	125 (46%)
Female	993 (56%)	42 (31%)	1037 (58%)	145 (54%)
Age				
< = 35	112(6%)	8(6%)	213(12%)	52 (19%)
35-60	1028 (58%)	56 (42%)	1065 (60%)	123 (46%)
>60	627 (35%)	70 (52%)	695 (39%)	95 (35%)
Annual income per capita (Yuan)#	5770	6794	6643	5824

Note: * X^2 ^= 31.02, P = 0.000; # Shandong, t = -3.79, P = 0.000; Ningxia, t = 3.15, P = 0.002

^i^Current member of New Co-operative Medical Scheme (NCMS)

^ii^Not currently a member of the NCMS

^iii^This refers to the total number of chronic disease cases reported. Some of the patients in the first row reported more than one chronic disease.

#### Prevalence of chronic diseases among rural residents in rural Shandong and Ningxia

Table 2 presents the prevalence of chronic diseases among the sample. In Shandong, the prevalence rate of one or more chronic diseases for NCMS members in 2005 was 16% computed by patients, which was higher than the prevalence rate for non-members (13%) and the rate at national level (10%). The total number of cases reported illustrates that some patients suffered from more than one chronic disease. A similar situation was found in Ningxia. Hypertension, heart diseases, gastroenteritis, and arthritis were the most common chronic diseases both in rural Shandong and Ningxia based on the number of cases. In Shandong, hypertension was the most common single disease with a prevalence rate of 4.49%, whilst in Ningxia, the arthritic diseases were the most common with a prevalence rate of 5.17%.

**Table 2:** Prevalence rate of chronic diseases in rural Shandong and Ningxia

Prevalence rate (%)	Shandong			Ningxia			National level (**%)**
		
	Total	NCMS	Non-NCMS	Total	NCMS	Non-NCMS	
Computed by patients	16	16	13	19	19	17	10
Computed by cases	20	20	16	26	27	22	12

*source: National Health Survey 2003

### Health care utilization by rural chronic disease patients in 2005-2006

#### Distribution of health facilities utilized by rural chronic disease patients

Table 3 presents the utilization of different levels of healthcare by chronic disease patients in the year prior to the survey. 11.9% of patients in Shandong and 10.7% in Ningxia did not seek any treatment. In both Shandong and Ningxia, the proportion of patients covered by NCMS who did not seek treatment was higher than that of non-members; this difference was significant in Ningxia but not in Shandong. However, it is important to note that the chronic disease prevalence rate was higher among NCMS members than non-members.

**Table 3:** Distribution of health facilities visited by rural patients with chronic diseases in Shandong and Ningxia

	**Shandong**		**Ningxia**	
	
	**NCMS**	**Non-NCMS**	**NCMS**	**Non-NCMS**
	
Total No. of chronically patients	1767	134	1773	270
No. of patients who did not seek any treatment	229(12.96%)*	14(10.45%)*	224(12.63%)#	21(7.78%)#
No. of patients visited village clinic	911(51.56%)	46(34.33%)	424(23.91%)	75(27.78%)
No. of patients visited Township health centre	432(24.45%)	23(17.16%)	456(25.72%)	70(25.93%)
No. of patients visited County Hospital	452(25.58%)	43(32.09%)	917(51.72%)	147(54.44%)
No. of patients visited drug stores	1076(60.89%)	98(73.13%)	1322(74.56%)	177(65.56%)

Note: *X^2 ^= 1.881, p = 0.170; # X^2 ^= 8.406, p = 0.004

Health-seeking behaviour by chronic disease patients differed between Shandong and Ningxia. In Shandong, over half of the patients sought care at drug stores regardless of NCMS membership status, and over half of NCMS members and a third of non members sought care at village clinics. In Ningxia, over half of the patients preferred to seek care at drug stores regardless of NCMS membership status, with the proportion visiting drug stores reaching 75% of NCMS members. However, over half of patients also sought care at the county hospital, regardless of NCMS status, in comparison to the 26% of NCMS members and 31% of non-members utilizing services at this level in Shandong.

#### Financial burden of chronic disease

Table 4 presents the proportion of chronic disease expenditure before reimbursement to annual per capita expenditure among chronic disease patients, divided by income quintiles. Overall, the proportion of families with NCMS membership spending over 40% of their annual non-food expenditure on treating chronic diseases in 2005-2006 was about 15% in Shandong and 14% in Ningxia, which was lower than the proportion of non-NCMS members (at 21% and 18% respectively) although the difference was not statistically significant in either province.

**Table 4:** Proportion of chronic disease expenditure to annual expenditure per capita [% (average chronic disease expense per capita/annual non-food expenditure per capita)]

Patients groups by income quintile	Shandong (%)		Ningxia (%)	
	
	NCMS	non-NCMS	NCMS	non-NCMS
Bottom 20%	45	122	44	56
20-40%	23	102	42	45
40-60%	30	23	45	35
60-80%	19	41	33	16
Top 20%	23	28	25	73
Average	27	47	35	42
The proportion of families whose health expenditure of chronic disease to non-food annual expenditure is over 40%*	15	21	14	18

* Shandong: X^2 ^= 2.76 p = 0.097; Ningxia: X^2 ^= 2.96 p = 0.085

For chronic disease patients covered by NCMS in Shandong, the average proportion of chronic disease expenditure to annual non-food expenditure was about 27%, which was slightly lower than that 35% in Ningxia. Among NCMS members, the poorest patients bore the heaviest financial burden of disease, with expenditure reaching 45% and 44% of annual non-food expenditure among the lowest income group in Shandong and Ningxia respectively, as compared to 23% and 25% for the highest income group.

In Shandong, in the poorest income group of non-NCMS members, the average proportion of chronic disease to annual non-food expenditure was extremely high at over 100%, due to one case in which a family spent nearly 60,000 Yuan(about 8000 US$, 1 US$ = 7.5 Yuan) on chronic disease treatment over the previous year. The high proportion in the second lowest income group was found for the same reason.

#### Extent of financial protection offered by NCMS to rural patients with chronic diseases

Table 5 presents the proportion of patients receiving reimbursement from NCMS and the level of that reimbursement. Generally in Shandong just under 50% of NCMS members with chronic diseases received any reimbursement in the previous year. Over half of the patients who ever visited village clinics or township health centers received reimbursement, but only 19% of patients seeking care at county hospitals, were reimbursed. Meanwhile in Ningxia, only 11% of NCMS members with chronic diseases received any reimbursement, with the highest proportion of patients reimbursed for visits to township health centers at 24%, compared with 2% and 9% of those seeking care at village clinics and county hospitals respectively.

**Table 5:** Proportion of chronic disease cases and expenditure at different health facilities reimbursed by NCMS

	Village clinic	Township health center	County hospital	Drug store	Total
**Shandong**					
No. of patients with chronic diseases reimbursed by NCMS	530 (58.24%)	236 (54.63%)	87 (19.25%)	0	853 (48.27%)
Medical expenditure (mean)	338.85	195.51	781.86	212.56	1528.77
Reimbursement (mean)	33.61	32.46	104.56	-	170.64
% of reimbursement to medical expenditure	9.92	16.60	13.37	-	11.16
**Ningxia**					
No. of patients with chronic diseases reimbursed by NCMS	7 (1.65%)	109 (23.90%)	82 (8.94%)	0	198 (11.17%)
Medical expenditure per capita (mean)	141.70	360.50	2012.11	458.45	2972.77
Reimbursement per capita (mean)	0.56	33.53	223.75	-	257.84
% of reimbursement to medical expenditure	0.39	9.30	11.12	-	8.67

The reimbursement rate was very low, with only 11% and 9% of overall medical expenditure for chronic diseases being reimbursed by NCMS in Shandong and Ningxia respectively. In Shandong, the reimbursement rate at the village clinic level was about 10% compared to a rate of 13% at the county hospital level. The highest rate of reimbursement was at the township health center, at nearly 17%, despite the low utilization of this level of services in the province. In Ningxia, expenditure at county hospital attracted the highest reimbursement rate out of the three levels of rural health care providers, but at a rate of only 11%.

The average expenditure at drug stores was 2 times higher in Ningxia 458 Yuan per capita per year) than Shandong (213 Yuan). No reimbursement was available for expenditure at drug stores in either province.

## Discussion

The first research question we aimed to investigate in this study was: what is the financial burden of chronic disease in the study areas, especially for the poor? Our findings show that healthcare expenditure for chronic diseases does place a heavy financial burden on many of those suffering from them, accounting for an average of 27% of annual non-food per capita expenditure amongst NCMS members in Shandong and 35% in Ningxia. Moreover, we found that around 14-15% of families in both Shandong and Ningxia spent more than 40% of their non-food expenditure on chronic healthcare costs.

International estimates of rates of 'catastrophic expenditure' vary greatly between countries and studies and comparability is limited by variations in methods. A 2003 multi-country analysis of catastrophic expenditure found that the proportion of households facing catastrophic payments from out-of-pocket health expenses varied widely between countries, from less than 0.01% in Czech Republic and Slovakia to 10.5% in Vietnam [32]. The proportion of households facing catastrophic expenditure due to chronic illness identified by our study is therefore relatively high. The definition of catastrophic payments we used was, however, conservative. Although our data may be subject to some recall bias, we used the highest threshold utilized by earlier studies. In addition, we did not include data on the indirect costs of seeking care, such as those associated with transport, food, and accommodation, which are often substantial in rural China [24] or lost earnings associated with time taken off work to seek care. Our estimate of the proportions of households facing catastrophic expenditure therefore probably underestimates the financial burden of chronic disease for households.

The potential impact of such levels of catastrophic expenditure on chronic disease treatment on the economy in rural China is heavy considering the estimated mortality rate of 10.5% [12]. For example, hypertension, a common chronic disease with a prevalence of about 19% nationally, affected an estimated 160 million patients in 2003, at a direct cost of 383.85 hundred million Yuan. Only 25% of patients are estimated to be receiving treatment, with a control rate of only 6%. Costs are likely to rise given increasing prevalence and if treatment rates improve [34].

The potentially catastrophic costs of seeking care may also have been a key reason for between 8 and 11% of non NCMS members and 13% of NCMS members not seeking any medical care for chronic illness in the previous year. Other studies have found that the poorest households who cannot afford 'catastrophic' healthcare expenditure' and may not be able to borrow money may simply opt not to seek care for their illness [33]. Leaving chronic conditions untreated may also lead to financial burdens for households due to the potential for worsening ill-health, which may reduce the ability of patients and their carers to earn income. For example, in China, long-term disability accounted for an estimated 43% of the indirect costs of hypertension, which totalled 6.11 billion Yuan in 2003 [24].

Our study found that the financial burden is heavier for the poorest, with a greater proportion of NCMS members in the poorest quintile facing catastrophic expenditure as compared to those in the richest quintile in both study sites. A number of those suffering from chronic disease suffer from more than one disease, which is likely to contribute towards the weight of the burden.

The second research question was: how far does NCMS offer financial protection against chronic disease burdens, especially for the poor? We found that only a relatively small number of NCMS members suffering from chronic diseases were able to get any of their healthcare costs reimbursed. Even where patients were able to get healthcare costs reimbursed, the level of reimbursement was relatively low, ranging from 10 to 13% in Shandong and from less than 1% to 11% in Ningxia. The low level of reimbursement is partly due to the focus of NCMS on offering financial protection against the costs of in-patient care. Although chronic disease costs in this study include in-patient care, a high proportion of cases sought care at village clinics and township health centres, which seldom provide in-patient care. Costs at these facilities may not be reimbursed (as in some counties in Shandong at village clinic level) or may only be reimbursed at a low level and only up to a (relatively low) fixed amount through the Family Account. The total annual allocation of 8 Yuan per person in the FA should be placed in the context of an average annual medical expenditure by chronic disease patients at the lowest level of healthcare of 142 Yuan (at the village level) in Ningxia and 196 Yuan (at the township level (in Shandong (see Table 5)). In Shandong, the level of reimbursement offered at the village clinic is especially low, despite the fact that the majority of patients utilize this service.

We found that a slightly higher proportion of non-NCMS member than NCMS member households faced catastrophic expenditure, which suggests that NCMS offers a degree of financial protection. However, the difference is relatively small and not statistically significant, suggesting the degree of protection is not great. In Ningxia, the financial protection effect appears to be lower for the poorest quintile than it is for the richest quintile since the difference between NCMS members and non-members is greater amongst the richest quintile than the poorest.

These findings have a number of implications for NCMS design and rural health policy. The design of the NCMS focuses on risk-pooling for in-patient costs, on the assumption that these are the costs most likely to prove catastrophic. However, our results show that a significant proportion of patients with chronic diseases do face potentially catastrophic healthcare costs and these are especially heavy for the poor. This is likely to be partly because a significant part of chronic disease expenditure occurs on out-patient care, for which very limited levels of reimbursement are available. However, the relatively low levels of actual reimbursement of in-patient expenditure may also contribute to the burden, especially where repeated hospital stays are required.

The proportion of chronic disease patients facing catastrophic health expenditure therefore poses a challenge to NCMS. China's NCMS is not alone in facing this challenge to the financial protection offered by health insurance. The degree to which social health insurance schemes should cover relatively low-cost (per unit) primary healthcare is debated internationally. It has been argued that other forms of subsidies such as equity funds and social cash transfers may provide more appropriate solutions for the most vulnerable than orienting the design of a whole insurance scheme towards 'low-cost' risk coverage [35]. Recent local policy developments in the NCMS in some counties and provinces have seen a shift away from the use of Family Accounts towards risk pooling for outpatient expenditure, which is a necessary, but not sufficient step to improve financial protection for chronically ill members. Changes in NCMS policy towards greater financial protection for the chronically ill will have substantial cost implications given the high, and increasing, prevalence of chronic disease. The central Chinese government has recently doubled its contributions to the NCMS in less economically developed provinces, and subsidies from government at all levels are planned to increase further over the next two years. The challenge is to ensure that NCMS design makes the best use of funds to prevent impoverishment and other aspects of health system policy play a role in controlling cost escalation and preventing and managing chronic disease. The current system places most of the burden of guarding against moral hazard on the patient side through low reimbursement rates, ceilings and deductibles, but there is a need to make more efforts to control moral hazards from the provider side.

The prevention of chronic diseases is an important area for health policy development to reduce the burden of ill health due to chronic illness [36] and therefore the overall costs of treatment for both households and the NCMS. Primary healthcare has an important role to play in managing chronic disease. Disease management programmes may also offer some advantages over the traditional acute, reactive, and episodic model of medical care for chronic diseases with regard to containing the costs of care, particularly by preventing unnecessary repeat diagnostic testing and expensive treatment due to avoidable complications [37,38]. The Chinese government should consider the inclusion of such programmes in the development of its medium and long-term national plans for chronic disease control and prevention.

## Conclusion

The heavy financial burden of healthcare for chronic disease and the proportion of chronic disease patients facing catastrophic health expenditure poses a challenge to NCMS. There is an urgent need for a clear policy on how to offer financial protection to those with chronic disease, which will require further, in-depth investigation of the burden of chronic illness.

Based on the current study results, it is recommended that a special benefit package for chronic diseases to enable increased levels of reimbursement for outpatient expenditure on chronic diseases should be designed and pilot-tested in the NCMS, and the impact on catastrophic healthcare expenditure for patients with chronic disease, especially the poor, should be assessed. The development of chronic disease management programmes should be considered as a complementary strategy.

## Competing interests

The authors declare that they have no competing interests.

## Authors' contributions

QS participated in data collection, conducted the analysis of the data, contributed to the interpretation of the data, and drafted the paper. XYL contributed to the design of the study, interpretation of the data and substantial critical revision of the paper. QYM contributed to the conception and design of the study, supervised data collection, and contributed to data analysis and interpretation. ST led the conception and design of the study and made commented on the substantial intellectual content of the paper. BRY contributed to study design, participated in data collection and contributed to data analysis and interpretation. RT contributed to the study conception and design, data interpretation, and paper drafting, and critically reviewed several versions for substantial intellectual content. All authors have read and approved the final manuscript.
